# Attention-Enhanced GAN for Spatial–Spectral Fusion and Chlorophyll-a Inversion in Chen Lake, China

**DOI:** 10.3390/s26072107

**Published:** 2026-03-28

**Authors:** Chenxi Zeng, Cheng Shang, Yankun Wang, Shan Jiang, Ningsheng Chen, Chengyu Geng, Yadong Zhou, Yun Du

**Affiliations:** 1 School of Geosciences, Yangtze University, Wuhan 430100, China; 2023710507@yangtzeu.edu.cn (C.Z.); ykwang@yangtzeu.edu.cn (Y.W.); 500053@yangtzeu.edu.cn (S.J.); chennsh@yangtzeu.edu.cn (N.C.); 2023710489@yangtzeu.edu.cn (C.G.); 2 Hubei Engineering Research Center of Unconventional Petroleum Geology and Engineering, Hubei Key Laboratory of Complex Shale Oil and Gas Geology and Development in Southern China, International Cooperation Center for Mountain Multi-Disasters Prevention and Engineering Safety, Yangtze University, Wuhan 430100, China; 3 Key Laboratory of Monitoring and Estimate for Environment and Disaster of Hubei Province, Innovation Academy for Precision Measurement Science and Technology, Chinese Academy of Sciences, Wuhan 430077, China; zhouyadong@apm.ac.cn (Y.Z.); duyun@whigg.ac.cn (Y.D.)

**Keywords:** Unsupervised GAN, Sentinel-2 MSI, Sentinel-3 OLCI, spatial–spectral fusion, Chlorophyll-a inversion

## Abstract

The Sentinel-3 Ocean and Land Colour Instrument (OLCI) is designed for water monitoring. Its 21-spectral bands serve as the basis for the precise retrieval of water quality parameters. However, its coarse resolution restricts the depiction of the spatial distribution of water quality parameters in small inland water bodies. Spatial–spectral fusion is a common method to address the inherent constraints between the spatial and spectral resolutions of sensors. Central to the popular methods is the deep learning-based method. Nonetheless, deep-learning-based models still face challenges in fusing Sentinel-2 Multi-Spectral Instrument (MSI) and Sentinel-3 OLCI data. Here, we propose a Multi-Scale-Attention-based Unsupervised Generative Adversarial Network (MSA-UGAN), which effectively integrates OLCI’s spectral advantage and MSI’s spatial resolution. Quantitative evaluation was conducted against five benchmark methods, including traditional approaches (GS, SFIM, MTF-GLP) and deep learning models (SRCNN, UCGAN). The results show that MSA-UGAN achieves the best overall performance: QNR (0.9709) and SSIM (0.9087) are the highest, while SAM (1.1331), spatial distortion (D_S_ = 0.0389), and spectral distortion (D_λ_ = 0.0252) are the lowest. This shows that MSA-UGAN can better preserve the spatial details of S2 MSI and the spectral features of S3 OLCI data. Moreover, ERGAS (2.2734) also performs excellently in the comparative experiments. The experiment of Chlorophyll-a inversion using the fused image in Chen Lake revealed a spatial gradient ranging from 3.25 to 19.33 µg/L, with the highest concentrations in the southwestern nearshore waters, likely associated with aquaculture. These results jointly indicate that MSA-UGAN can generate high-spatial-resolution multispectral images, and the fused images can be effectively utilized for water quality monitoring, thereby providing essential data support for the precision management and scientific decision-making regarding inland lakes.

## 1. Introduction

With the intensification of urbanization activities, the issue of eutrophication in inland lakes has become increasingly severe [1]. It not only threatens regional drinking water safety but also poses a significant challenge to the sustainable development of the ecological environment and socio-economic systems [2,3]. To effectively address the challenges of water eutrophication, a comprehensive understanding of water quality is the primary prerequisite, as it offers a scientific basis and guidance for eutrophication control. The key to comprehensively understanding water quality lies in the precise monitoring of various water quality parameters. Traditional water quality monitoring methods, which rely on manual field sampling and laboratory analysis, are often time-consuming, labor-intensive, and costly. In contrast, remote sensing technology has emerged as an efficient alternative due to its high temporal and spatial resolution, low cost, and its ability to effectively characterize the distribution and temporal variations in large-scale water bodies [4].

Currently, Sentinel-3 Ocean and Land Color Instrument (S3 OLCI) is widely used in water quality research for oceans and large water bodies due to its design-specific advantages and excellent spectral performance, offering 21 spectral bands (Oa1–Oa21) with high spectral resolution and narrow bandwidth (~10 nm) across the visible to near-infrared spectrum (390–1040 nm) [5,6]. However, its 300 m spatial resolution is generally regarded as too coarse for most inland waters [7]. These inland lakes are numerous, widely distributed, and relatively small in area, which makes it difficult to accurately delineate the water-land boundary at the pixel scale. When monitored using satellite images of low spatial resolution, a single pixel often contains both water and land information, thus making it difficult to accurately delineate the water-land boundary. The limited spatial resolution of such images often leads to the phenomenon of mixed pixels. Specifically, the spectral signal of the water body is influenced by the adjacent land, which significantly reduces the accuracy of water remote sensing reflectance—an essential prerequisite for deriving key parameters such as Chlorophyll-a (Chl-a) concentration and turbidity [8]. Consequently, the limited spatial resolution of S3 OLCI significantly increases the difficulty and uncertainty of retrieving water quality parameters from mixed pixels, restricting its applicability in the assessment of eutrophication in most small inland lakes.

In contrast to S3 OLCI, the Sentinel-2 Multispectral Instrument (S2 MSI) provides high spatial resolution, with a ground sampling distance ranging from 10 to 60 m, enabling detailed observation of inland water bodies. Although the improved spectral range (443–2190 nm) and 13 spectral bands (B1–B12) significantly enhance its ability to obtain water quality information [9,10], the S2 MSI is primarily designed for land observation. Its spectral observation capability is limited when compared to that of the S3 OLCI, which mainly focuses on water and atmospheric observations. This limitation can be summarized as a relatively smaller number of bands, the band configuration, and a lower signal-to-noise ratio compared to the S3 OLCI. These limitations result in the absence of certain wavelengths that are crucial for water quality monitoring, such as the Chl-a absorption peak at 665 nm, thus directly affecting the accuracy of water quality parameter inversion [11]. Addressing the issue of the spatial and spectral interdependence of sensors, it is an intriguing topic to consider how to provide remote sensing images that simultaneously possess high spatial and spectral resolution for high-precision water quality monitoring.

One approach to address the fundamental constraint between a sensor’s spatial and spectral resolutions is spatial–spectral fusion. This fusion process is to combine the high spatial resolution of S2 multispectral images with the spectral information from the OLCI on S3, thereby producing higher spatial resolution multispectral (HRMS) images [12]. From traditional algorithms to the most recent deep learning (DL)-based algorithms, the spatial–spectral fusion methods proposed in the literature can be primarily categorized into four main types: component substitution (CS) [13,14,15,16], multi-resolution analysis (MRA) [17,18], multi-scale decomposition (MSD) [19,20], and DL [21,22,23]. Each type has distinct strengths and limitations. CS methods enhance spatial resolution by replacing the intensity component of hyperspectral images (HSI) with high-spatial-resolution images. Essentially, it is a linear transformation under the hypothesis of spectral-spatial decoupling. Their main advantage lies in computational simplicity and sharp spatial enhancement, making them suitable for applications where processing speed is prioritized. Representative methods of this type include the Gram-Schmidt transformation, PCA, and the Brovey algorithm [13,16,24]. However, they may introduce notable spectral distortion due to inherent differences between components [25]. This is particularly evident in water pixels, where the subtle variations in water spectral characteristics are highly susceptible to the amplification of spatial interpolation errors during the intensity replacement process. This process is influenced by complex factors such as strong adjacency effects and heterogeneous atmospheric scattering, which results in systematic biases in the inversion results [26]. MRA methods, conversely, inject high-frequency spatial details from high-spatial-resolution images into the HSI. They are widely acknowledged for attaining superior spectral preservation compared to CS methods and have demonstrated effectiveness in numerous land-cover applications where upholding spectral fidelity is crucial. Representative methods of this type include algorithms based on the Laplacian pyramid and algorithms based on wavelet transforms, among others [17,18]. Nevertheless, they are more prone to spatial distortion and may not fully capture low-frequency differences [27]. Particularly in S2 MSI and S3 OLCI cross-scale fusion, due to the mismatch between the spectral response functions of OLCI’s 21 narrow bands and the MSI broadband, the MRA methods are highly vulnerable to the risk of band aliasing [28]. MSD methods aim to estimate and separate the spatial-structural and spectral-attribute components from input images. The core idea involves decomposing the high-spectral-resolution HSI to extract a representative spectral signature matrix, while simultaneously decomposing the high-spatial-resolution multispectral images to derive a detailed spatial structure matrix. The final fused images are then reconstructed by integrating the spectral matrix from the HSI with the spatial matrix from the multispectral images. Representative methods of this type include algorithms based on curvelet transform and algorithms based on contourlet transform, etc [19,20]. This approach better preserves both spatial and spectral information but often requires complex optimization processes, which could affect model robustness and computational efficiency [23].

In recent years, DL algorithms have emerged as powerful feature extractors in computer vision and have been widely adopted for image fusion tasks [21,29]. The DeepSen3 model proposed by Alboody et al. [30] is the first to introduce a multi-scale residual Inception convolutional network into the fusion task of S2 MSI and S3 OLCI. By designing a multi-scale feature extraction module and a residual learning mechanism, it aims to enhance spatial resolution while preserving spectral information as much as possible. Experimental results indicate that DeepSen3 significantly outperforms existing deep learning methods such as SSR-NET and MSDCNN in quantitative metrics (PSNR, SAM, ERGAS). Nevertheless, DeepSen3 still has certain limitations: its loss function is based on the mean squared error (MSE), lacking explicit constraints on spectral physical curves. Moreover, its multi-scale module primarily focuses on spatial feature extraction and does not establish an explicit decoupling mechanism for the nonlinear coupling and mutual interference between spectral features and spatial features. The authors also point out in the conclusion that introducing generative adversarial networks to further enhance the fusion quality is an important direction for future research. The pix2pix framework proposed by Isola et al. [31] is a pioneering application of conditional generative adversarial networks in image-to-image translation tasks. This framework achieves end-to-end mapping from input to output by jointly employing adversarial loss and pixel-level loss (Ɩ_1_), and utilizing the U-Net and PatchGAN architectural designs. Although pix2pix was not originally designed for remote sensing imagery, its supervised learning paradigm and feature extraction-fusion-reconstruction framework have provided important insights for subsequent image fusion research. However, supervised GANs typically require HRMS images to be simulated via synthesis or degradation processes. Therefore, their performance often depends on the quality of the training samples. In practical remote sensing scenarios, ideal HRMS reference images are often difficult to acquire, which restricts the application of supervised GAN models in most real-world situations. To address this limitation, Zhou et al. [32] proposed the Unsupervised Cyclic-consistent Generative Adversarial Network (UCGAN). By introducing a cycle-consistency mechanism, this model can be trained directly on original-scale images without the need for any synthesized HRMS images. It also incorporates a dual-stream generator to extract modality-specific features from PAN and MS images and utilizes a spatial–spectral reconstruction loss to ensure the fidelity of the fusion results. Experiments demonstrate that UCGAN significantly outperforms supervised methods in fusion quality on full-scale images. However, UCGAN is primarily designed for panchromatic sharpening tasks, with inputs being panchromatic images and low-resolution multispectral images. When the task extends to heterogeneous sensor fusion, such as between S2 MSI and S3 OLCI, which exhibit significant differences in spectral resolution and greater spatial scale variations, effectively decoupling spectral features from spatial textures and preventing mutual interference of features in an unsupervised framework remains an important problem that needs to be addressed.

Therefore, the primary challenges encountered by existing DL-based fusion methods when performing spatial–spectral fusion of S2 MSI and S3 OLCI images for water quality monitoring tasks can be summarized as follows:

Firstly, directly performing pixel-level fusion between S2 MSI and S3 OLCI may cause mutual interference between spatial and spectral features [33,34]. In the application presented in this paper, this phenomenon is mainly manifested in two types of feature degradation: (i) Spatial features (such as water texture boundaries) are misrecognized as spectral noise and suppressed, leading to over-smoothed edges in the fused images. (ii) During the process of injecting spatial details, spectral features are distorted, causing color shifts in water pixels and affecting subsequent inversion tasks. In short, this mutual interference between spatial features and spectral features makes it difficult for the network to effectively distinguish and independently optimize these two types of features, ultimately leading to subpar fusion quality [35,36,37].

Secondly, most GAN-based spatial and spectral fusion models tend to focus on improving the visual quality of the fused results or the similarity of spatial structures, while lacking explicit constraints on the spectral physical dimension. This may result in fused images that, while retaining clear textures, exhibit shifted spectral curves, thereby compromising the original radiometric characteristics of the objects. Especially in water quality retrieval tasks, the spectral fidelity of the fused results is crucial [38].

Third, although GANs have achieved remarkable progress in the field of remote sensing image fusion, most related studies focus on tasks such as panchromatic sharpening or infrared-visible fusion. Concerning cross-sensor fusion issues like S2 MSI and S3 OLCI, only a few CNN-based methods, such as DeepSen3, have been reported in the literature, while GAN-based research on S2 MSI and S3 OLCI fusion remains limited.

In view of these challenges and the advantages and potential of adversarial learning in feature extraction and prediction tasks [39], this work aims to bridge the spatial and spectral gaps between S2 MSI and S3 OLCI through a novel spatial–spectral fusion framework (MSA-UGAN). Our key contributions are as follows:

1. In our dual-stream network, we redesigned the spatial and spectral subnetworks, physically isolating the extraction paths of S2 MSI spatial features and S3 OLCI spectral features to prevent interference between different modalities at the early stages. Additionally, we introduced a cross-attention mechanism to optimize cross-modal interaction, avoiding the direct concatenation of the extracted dual-stream features. Such direct concatenation could lead to forced mixing of modal features and information conflicts during the fusion stage, thereby causing cross-modal feature interference.
2. We designed a Multi-scale Attention-based Unsupervised GAN (MSA-UGAN) for the spatial–spectral fusion of S2 MSI and S3 OLCI data. The model incorporates diverse multi-scale modules and attention mechanisms. These are designed to enable the generative network to comprehensively capture and learn detailed features and spectral information across different spatial resolutions, thereby enhancing the spatial and spectral fidelity essential for water quality inversion tasks.
3. We designed an improved downsampling-consistent spectral angle loss based on the practical requirements of water quality inversion applications, aiming to optimize and constrain spectral features, thereby further ensuring the reliability of water quality inversion results.
4. We applied fused images to the inversion of Chl-a concentration in Chen Lake. Through the synergistic inversion of S2 MSI data, we evaluated the spectral advantages and application potential of fused images for monitoring Chl-a concentration in inland lakes. Experimental results show that fused images generated by MSA-UGAN can provide reliable data support for inland lake monitoring tasks.

The remainder of this paper is structured as follows. Section 2 introduces the study area, the materials required for spatial–spectral fusion, and the method for retrieving Chl-a concentration. Section 3 introduces the detailed architecture of the MSA-UGAN, including the generative network, the discriminative network, and the joint loss function. Section 4 presents a comparative analysis of our method with several classical fusion techniques and discusses the experimental results. Additionally, a spectral fidelity analysis was carried out. Section 5 presents the application of Chl-a concentration retrieval in Chen Lake based on the fused image, and further validates the fusion effect through collaborative inversion using the S2 MSI image. Finally, Section 6 discusses the limitations of this study, and Section 7 provides a summary of the paper.

## 2. Materials

### 2.1. Study Area

**Figure 1:**
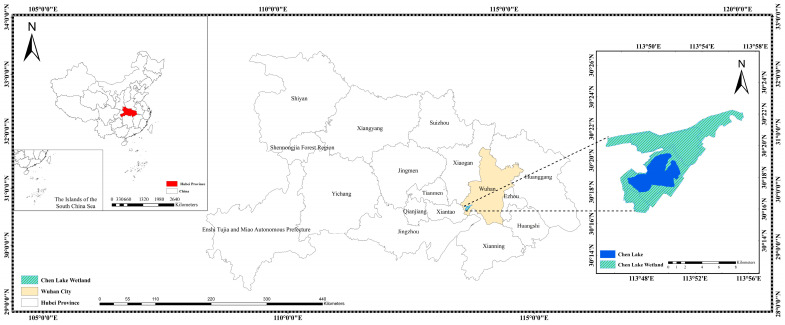
The geographical location of Chen Lake Wetland and Chen Lake in Wuhan, China. In the map, the red polygon on the left indicates the location of Hubei Province within China. The light orange polygon in the center shows the location of Wuhan City within Hubei Province and the green polygon represents the location of Chen Lake Wetland in Wuhan. On the right, Chen Lake is marked in blue within the green area of Chen Lake Wetland.

The Chen Lake Wetland is situated at approximately 113°46′–113°56′ E and 30°16′–30°23′ N, located in the delta area where the Yangtze River and the Han River meet, within the southwestern part of Caidian District, Wuhan (see Figure 1). It is primarily composed of Chen Lake, Zhangjia Grand Lake, Wangjia She Lake, and parts of the flood detention area of Dujia Tai, with a total area of 11,579.1 hectares. Chen Lake Wetland is not only the largest typical freshwater lake marsh wetland in the Jianghan Plain but also a crucial stopover along the East Asia-Australasia bird migration route. As a key ecological node in the Yangtze River Basin, it plays a vital role in maintaining regional biodiversity and water security. Therefore, conducting accurate and high-resolution monitoring of its water quality is both ecologically significant and methodologically valuable.

### 2.2. Data Preparation and Preprocessing

#### 2.2.1. Resolution Discussion

Given that the native 300 m resolution of OLCI data presents a 30-fold gap with the 10 m MSI bands and a 15-fold gap with the 20 m MSI bands, directly upscaling them to 10 m and 20 m would present an ill-posed learning problem for the fusion model. In this scenario, a single low-resolution pixel corresponds to an excessive number of high-resolution pixels, making the mapping learned by the model highly uncertain and ambiguous, and prone to mode collapse or spectral distortion artifacts. The experimental results of testing with UCGAN [32] indicate that when the preprocessed 10 m and 20 m MSI data are used as model inputs along with 300 m resolution OLCI data, excessively large scaling factors significantly compromise the stability of the training process, resulting in poorer fusion quality. Therefore, our framework opts for a 60 m resolution for fusion. This strategy strikes a balance between information retention and learning difficulty: compared to the coarse 300 m resolution, the 60 m resolution provides richer local textures and edge features for high-precision water quality monitoring, offering essential guidance for the model to reconstruct high-frequency details. Additionally, the selection of this spatial resolution is also a trade-off based on test results and application requirements. Although it inevitably sacrifices the higher spatial resolution of S2 MSI, it has been shown to effectively mitigate issues such as spectral aliasing and the generation of spatial artifacts caused by large scale factors (30×/15×), thereby ensuring the stability of the fusion process and the reliability of water quality retrieval results.

#### 2.2.2. Satellite Data Preprocessing

The remote sensing data sources used in this study include 20 pairs (amounting to 40 images) of S2 MSI Level-2A (L2A) and S3 OLCI Level-1B (L1B) products. These products were obtained from the European Space Agency (ESA) official website (https://browser.dataspace.copernicus.eu/, accessed on 20 June 2025), covering the same observation periods and overlapping geographic areas. This dataset comprises four scenes per year for the specific study area in Wuhan, captured between 2021 and 2025. The acquisition dates are as follows: 12 January 2021; 9 May 2021; 30 August 2021; 13 November 2021; 26 February 2022; 7 April 2022; 15 August 2022; 19 October 2022; 7 April 2023; 5 August 2023; 23 November 2023; 23 December 2023; 11 February 2024; 19 March 2024; 4 August 2024; 7 November 2024; 1 January 2025; 11 January 2025; 1 May 2025; 11 May 2025.

We referred to the multi-source remote sensing image registration approach in TDGAN [23]. The specific image preprocessing and alignment steps are as follows. First, the S3 OLCI L1B products were geometrically corrected using the UTM-WGS 1984 system to unify them with the geographic coordinate system of S2 MSI images. Subsequently, atmospheric correction was performed using the Case 2 Regional CoastColour (C2RCC) algorithm integrated into SNAP. This algorithm was proposed by Brockmann et al. [40] and has been optimized for optically complex Type II waters. It has now been extensively validated in remote sensing applications for inland and nearshore waters [41]. As the S2 MSI L2A products are already surface reflectance data that have undergone geometric correction, radiometric calibration, and atmospheric correction, no additional preprocessing is required. This study selected only the 9 bands of S2 MSI that overlap with the spectral range of S3 OLCI (B1-7, B8A, B9) for the fusion experiment. This is because, in the fusion experiment, we focus on the spatial resolution of S2 MSI. Since the originally downloaded S2 MSI files already contain B1-7, B8A, and B9 as surface reflectance data at a 60 m resolution, we only integrated the 60 m resolution data of the 9 bands in S2 MSI into a multispectral image for further processing.

Subsequently, the S2 MSI was used as a reference to perform sub-pixel registration of the S3 OLCI data. Following a hierarchical registration strategy [42], we first performed initial geometric alignment based on the geolocation information in the product metadata. Subsequently, a dense grid of tie points was generated using a moving window method (window size: 64 × 64 pixels). Normalized cross-correlation (NCC) maximization was used as the similarity measure [43] to determine the initial integer-pixel offset. Then, the initial pixel-level offset was refined to sub-pixel accuracy using a method based on Fourier domain phase correlation [44]. To eliminate mismatches potentially caused by cloud coverage, land-water boundaries, or seasonal changes in surface features, a random sample consensus (RANSAC) algorithm [45] was further applied to filter the tie points and fit a first-order polynomial transformation model. The final registration accuracy is evaluated through the root mean square error (RMSE) of independent check points, with the error controlled at less than one pixel to ensure spatial alignment accuracy prior to fusion. After registration, both datasets were clipped according to the effective spatial coverage of the S2 MSI images, resulting in a pair of spatially aligned images.

#### 2.2.3. Fusion Dataset Preparation

To mitigate the risk caused by the inherent challenge of mismatched spectral response functions (SRFs) between S2 MSI and S3 OLCI data, we first convolved and resampled the 9-band S2 MSI L2A data to match the center wavelengths and bandwidth definitions of the 21 S3 OLCI bands. This step aims to unify the spectral definitions of input data, enabling the subsequent dual-stream feature extraction network to focus on the extraction of spatial and spectral features, without the need to additionally learn the complex spectral mapping from 9 bands to 21 bands. This step utilized the official SRF curves provided by ESA [46,47] and was completed using a linear spectral resampling model [23,48]. Thereby, it generated S2_S3_SRF_ images (21 bands, 60 m) whose spectral characteristics were similar to those of S3 OLCI. Subsequently, we randomly cropped 320 × 320 pixel image patches from the S2_S3_SRF_ images. According to a spatial scale ratio of 5:1 (300 m:60 m), corresponding 64 × 64 pixel image patches were cropped from the S3 OLCI images covering the same physical areas, ensuring that all cropped regions are aligned in geographic coordinates. After cropping, to avoid the impact of differences in value ranges between the two data sources on training stability, we calculated the statistical features (mean and standard deviation) of the training set of each data source separately and performed normalization accordingly.

We split the dataset in an 8:2 ratio, with the training set comprising 256 blocks randomly selected from the remote sensing images, and the test set comprising 62 randomly selected blocks. To mitigate over-fitting and improve the model’s generalization performance, we employed data augmentation techniques on the 256 training patches, thereby expanding the training set to 2048 samples to ensure a sufficient evaluation basis. The data augmentation pipeline incorporated a series of geometric transformations applied stochastically during training. Each training patch was subjected to horizontal flipping with a probability of 0.5, vertical flipping with a probability of 0.5, and a random rotation selected from [0°, 90°, 180°, 270°]. This augmentation strategy effectively expanded the original dataset by a factor of 8 (accounting for 2 flipping directions × 4 rotation angles). The final training dataset was scaled to 2048 samples using these augmented variants (see Table 1).

### 2.3. Parameter and Algorithm Selection for Water Quality Retrieval

Chl-a is a key photosynthetic pigment in phytoplankton, and its concentration is strongly correlated with the biomass of phytoplankton. The excessive proliferation of phytoplankton is one of the direct manifestations of water eutrophication. Thus, elevated Chl-a levels typically signal eutrophication and even ecosystem degradation, making it a critical parameter for assessing eutrophication [49,50,51]. Therefore, accurate quantification of Chl-a concentration is essential in lake water quality monitoring. From a technical perspective, Chl-a concentration is currently one of the most mature and reliable parameters retrievable via water color remote sensing. This maturity allows us to fully utilize the fused images derived from S2 MSI and S3 OLCI data, providing a feasible method for rapidly and macroscopically characterizing the spatial distribution of lake eutrophication. Based on these considerations, this study selects Chl-a concentration as the key indicator for remote sensing-based water quality inversion in Chen Lake.

To verify the effectiveness of the fused images in water quality monitoring, we extracted the spectral reflectance values of the fused images at each sampling point in Chen Lake. Combined with in situ measured Chl-a concentration data, the Pearson correlation coefficient method [52] was employed to analyze the correlation between the measured Chl-a concentrations in Chen Lake and the corresponding reflectance values of individual bands and band combinations from the contemporaneous fused images. Based on the bands and band combinations that are highly correlated with Chl-a concentration, three representative machine learning algorithms—Support Vector Regression (SVR) [53], Random Forest (RF) [54], and Extreme Gradient Boosting (XGBoost) [55]—were selected to construct the Chl-a concentration inversion models in Chen Lake. Ultimately, a spatial distribution map of Chl-a concentration in Chen Lake was generated based on the optimal Chl-a inversion model.

### 2.4. Evaluation Indicators

To evaluate the performance of the proposed MSA-UGAN, three non-reference metrics commonly used in multispectral image fusion algorithms were employed to quantitatively evaluate the fusion results: Spectral Distortion (D_λ_), Spatial Distortion (D_S_), and Quality with No Reference (QNR) [56]. Additionally, three reference metrics were used: spectral angle mapping (SAM) [57], the relative dimensionless global error in synthesis (ERGAS) [58], and the structural similarity index (SSIM) [59] to quantitatively assess the fusion results.

QNR is a comprehensive no-reference quality metric, computed using both D_λ_ and D_S_, and is utilized for the overall evaluation of fusion quality. A higher QNR value (the best value being 1) signifies a better balance between spectral fidelity and spatial resolution in the fused result. Specifically, D_λ_ assesses the preservation of spectral information by calculating the differences between the fused image and the input S3 OLCI image across spectral bands, with a lower value (the best value being 0) indicating better spectral preservation. D_S_ evaluates the similarity of high-frequency spatial details between the fused image and the input S2_S3_SRF_ image by comparing the spatial detail features. A smaller D_S_ value (the best value being 0) suggests that the fused image retains more spatial details from the S2_S3_SRF_ image. SSIM measures the structural similarity between the fused image and the reference image by comparing luminance, contrast, and structural information, with values closer to 1 indicating higher structural fidelity. ERGAS evaluates the spectral fidelity of multispectral images by integrating the root mean square error of each band with the resolution ratio, where values closer to 0 indicate lower spectral distortion. SAM quantifies the degree of spectral distortion by calculating the angle between two spectral vectors, with values closer to 0 representing better spectral similarity. All experiments were repeated five times with different random seeds, and the final scores for each metric were reported as mean ± standard deviation.

To assess the accuracy of each Chl-a concentration inversion model, we selected the coefficient of determination (R^2^), root mean square error (RMSE), and mean absolute error (MAE) as the primary indicators for evaluating model performance. Among these, RMSE quantifies the disparity between the true and predicted Chl-a concentrations, where lower values correspond to higher inversion accuracy. R^2^ reflects the goodness-of-fit between the true and predicted values, with higher values (closer to 1) indicating a stronger correlation. MAE, as an absolute measurement indicator, represents the average absolute difference between the true and predicted Chl-a concentrations, where smaller values denote a better model fit.

It should be noted that in fields such as water quality inversion, the indicators RMSE and MAE are mainly used for relative comparisons between models. At present, there is no unified RMSE or MAE threshold applicable to all water bodies. Their acceptable ranges are highly dependent on the specific water quality conditions and research context. For example, the magnitude of Chl-a concentrations differs between eutrophic lakes and clean reservoirs, and consequently, the ranges of RMSE and MAE will also differ.

## 3. Methodology

### 3.1. Overview of MSA-UGAN

In general, Multiscale Attention-based Unsupervised GAN (MSA-UGAN) consists of two parts: a generative network *G*_1_ and a discriminative network *D*_1_. It should be clearly stated that all the attention mechanisms and multi-scale modules cited in this paper are progressive optimizations specifically for the fusion task of S2 MSI and S3 OLCI heterogeneous remote sensing images. They are targeted improvements based on the UCGAN model, rather than entirely novel attention fusion architectures.

**Figure 2:**
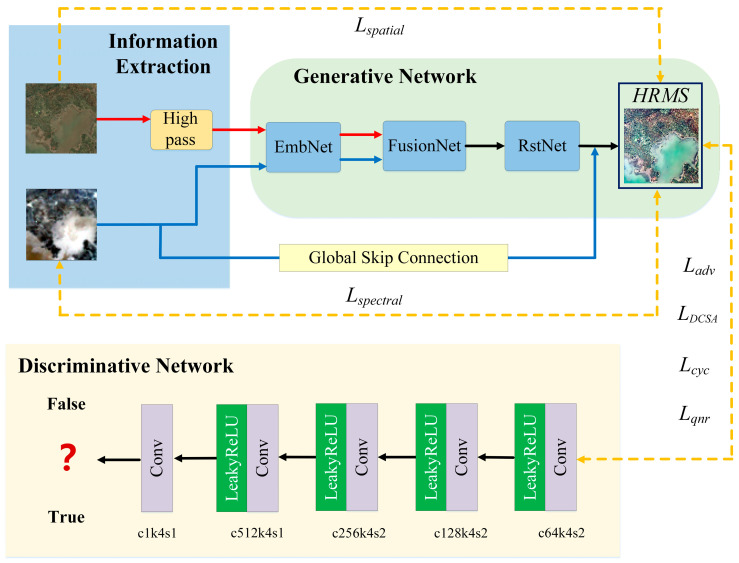
Overview of the proposed MSA-UGAN framework. “High Pass” is a filter used to extract high-frequency information from an image. The solid line represents the data flow. The red solid line represents the features from S2_S3_SRF_, the blue solid line represents the features from S3 OLCI, and the black solid line represents the features after the fusion of both types of features. The dashed line represents loss calculation. The blue background represents “Information Extraction” and the green background represents the “Generative Network”. The light yellow background represents the “Discriminative Network”, which shows the detailed architecture of *D*_1_. *D*_1_ follows the PatchGAN architecture and consists of five convolutional layers. The LeakyReLU activation function is used in *D*_1_. “c64k4s2“ defines a convolutional layer with a kernel size (*k*) of 4 × 4, a stride (*s*) of 2, and an output channel (*c*) of 64. There is a global skip connection from the original S3 OLCI to the final output, aimed at stabilizing the network and facilitating training.

The model input consists of the S2_S3_SRF_ (320 × 320 × 21_SRF_), which is a high spatial resolution S2 MSI image simulated through the SRF to match the spectral response function of S3 OLCI, as well as the original S3 OLCI image (64 × 64 × 21). The output is the HRMS image (320 × 320 × 21). Its overall framework is illustrated in Figure 2. Under the unsupervised framework, the overall authenticity of the model relies entirely on the mutual validation of the input data itself, without the need for synthetic or degraded HRMS images as supervisory information. The generative network employs a dual-stream feature extraction-fusion-reconstruction architecture, where the high-resolution S2_S3_SRF_ image and the low-resolution multispectral S3 OLCI image are mapped into the feature domain for fusion and then reconstructed back into the image domain. The discriminative network guides the training of the generative network by assessing the consistency between the generated fused features and the original input data at both spatial and spectral information levels. Additionally, the improved hybrid loss function jointly constrains and balances the recovery of spatial details and the preservation of spectral information under unsupervised conditions.

Our *G*_1_ comprises three components: a dual-stream feature embedding network (EmbNet), a multi-scale attention fusion network (FusionNet), and a reconstruction network (RstNet). To enhance the spatial details of the fusion results, we first apply high-pass filtering to the S2_S3_SRF_ images to extract their high-frequency information. Specifically, following the approach of UCGAN [32], we use an averaging filter to obtain the low-frequency component of the images. Then, we subtract this low-frequency component from the original images to obtain the high-frequency component representing edges and textures. Thereafter, the high-frequency information extracted from the S2_S3_SRF_ images and the normalized OLCI tensors will be processed through a dual-stream feature extraction network, whose two subnetworks respectively focus on the spatial information of the S2_S3_SRF_ and the spectral information of the OLCI, mapping these features into a higher-dimensional feature space and outputting the corresponding embedded feature vectors. Before inputting into FusionNet, we upsample the low-resolution S3 OLCI feature maps to match the spatial dimensions of the S2_S3_SRF_ feature maps by using bilinear interpolation combined with 1 × 1 convolution. Subsequently, the embedding vectors extracted by the two sub-networks are input into FusionNet for feature fusion, ultimately generating an integrated embedding vector. Finally, RstNet transfers the embedding vector back to the image domain to produce the fused HRMS images with 21 bands. *D*_1_ adopts the PatchGAN architecture [31]. Its details can be obtained in Figure 2. Additionally, to enable the model to effectively preserve the spatial and spectral information of the original data under an unsupervised framework and to enhance the reliability of the fused images in water quality retrieval, we optimized the constraints within the framework. The hybrid loss function includes the downsampling-consistency-based spectral angle loss (*L_DCSA_*), adversarial loss (*L_adv_*), reconstruction loss (*L_rec_*), cycle consistency loss (*L_cyc_*), and no-reference loss (*L_nrf_*). The specific details of these loss functions will be discussed in detail below.

### 3.2. Generative Network of MSA-UGAN

#### 3.2.1. Embedding Network

**Figure 3:**
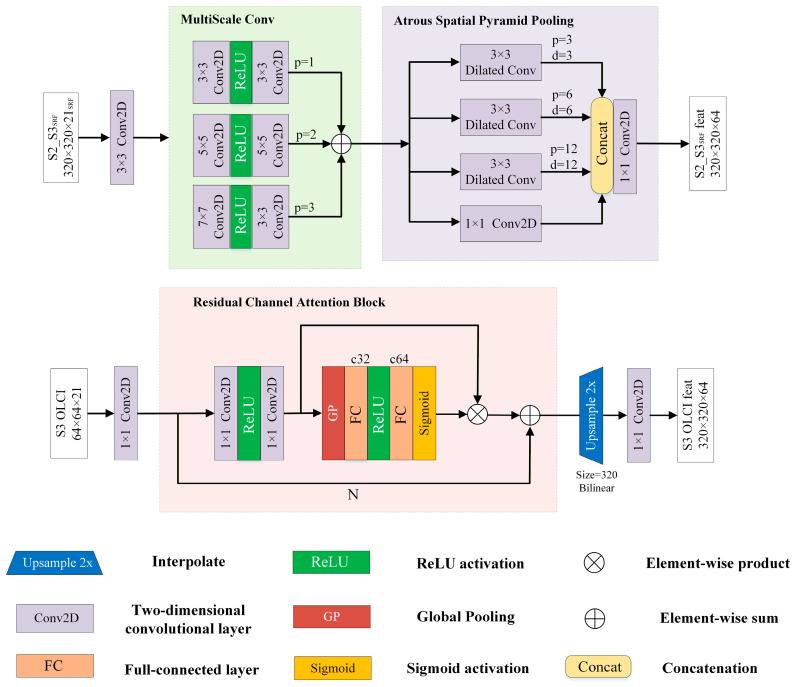
Detailed architecture of EmbNet. The diagram details the layer parameters. ‘*d*’ stands for Dilation, and ‘*p*’ stands for Padding. For layers where the stride is not specified, it defaults to 1, and the number of output channels defaults to 64.

To reduce potential inter-feature interference caused by resolution mismatches between S2_S3_SRF_ and S3 OLCI inputs, EmbNet employs a dual-stream network to independently extract spatial features (S2_S3_SRF_) and spectral features (S3 OLCI) respectively (Figure 3). The spatial subnetwork of EmbNet includes a multi-scale convolution module (MultiScaleConv) as well as an Atrous Spatial Pyramid Pooling (ASPP) [60]. First, the input data is initially subjected to a 3 × 3 convolutional layer for preliminary feature extraction and dimensional adjustment, mapping the S2_S3_SRF_ inputs into the feature space. Subsequently, MultiScaleConv captures spatial features at different scales by using convolution kernels of varying sizes (3 × 3, 5 × 5, 7 × 7) in parallel, and these features are fused through summation [61]. However, in remote sensing images, the scales of objects often varied significantly, and relying solely on MultiScaleConv made it difficult to fully capture the global contextual information in complex scenes. Therefore, we introduced ASPP to meet the requirements of a large-scale receptive field. ASPP could enhance the network’s capability to perceive large-scale spatial structures by constructing multi-scale receptive fields through parallel atrous convolution branches with different dilation rates. Specifically, the ASPP module consisted of three parallel atrous convolution branches with dilation rates of 3, 6, and 12, each employing a 3 × 3 convolution kernel, which could expand the receptive field efficiently without increasing the number of parameters. Moreover, excessively large dilation rates might cause the sampling points of the convolution kernel to become too sparse, leading to the loss of local features. Therefore, a 1 × 1 standard convolution branch was introduced to preserve features at the original scale, ensuring the network’s sensitivity to small objects. Subsequently, the output features from the four branches were concatenated along the channel dimension and then reduced in dimensionality through a 1 × 1 convolution layer, ultimately producing feature vectors rich in multi-scale spatial contextual information.

Since the channel dependence of spectral flow is globally strongly correlated, a single-layer channel attention mechanism is sufficient for modeling, eliminating the need for redundant computations involving multi-layer spatial convolutions and channel fusion. Therefore, in order to focus on the extraction of spectral information from S3 OLCI images, we introduce a single simplified residual channel attention block (RCAB) [32] in the spectral branch to concentrate on the extraction of spectral features. Specifically, the initial 1 × 1 convolutional layer first maps the S3 OLCI input into the feature space. The simplified RCAB performs preliminary feature extraction on the input feature map through 1 × 1 convolution operation. Subsequently, average pooling compresses the spatial information of each channel in the convolved feature map into a scalar, which is then processed by a Multi-Layer Perceptron (fully connected layer with activation function) to generate channel attention weights. These weights are multiplied with the convolved feature map channel-wise, adaptively enhancing key channels while suppressing noise. Finally, the input feature map is combined with the weighted features through a residual connection, enabling feature optimization and stabilizing the network training process.

Before inputting into the FusionNet, we performed a scale transformation on the S3 OLCI data in the feature domain to align it with the 320 × 320 feature vectors extracted from the spatial flow in the spatial dimension. This was done in order to avoid potential issues that may arise from directly sampling in the image domain. Specifically, bilinear interpolation is first used to upsample the S3 OLCI feature vectors from the 64 × 64 low resolution to the target resolution of 320 × 320. This method of center alignment is used to ensure spatial continuity. Bilinear interpolation, as a low-pass filtering operation, generates new pixels via neighborhood-weighted averaging, enabling feature maps to be enlarged without introducing high-frequency noise. To optimize the quality of upsampled features, a 1 × 1 convolutional layer is added after the upsampling layer. This convolutional layer does not alter the spatial resolution and is intended to integrate multi-channel information, achieving linear recombination and enhancement of feature channels. By carrying out scale transformations in the feature domain and incorporating the upsampling operation as part of the network for end-to-end training, it provides an adaptive spatial alignment foundation for subsequent feature fusion.

#### 3.2.2. Fusion Network

**Figure 4:**
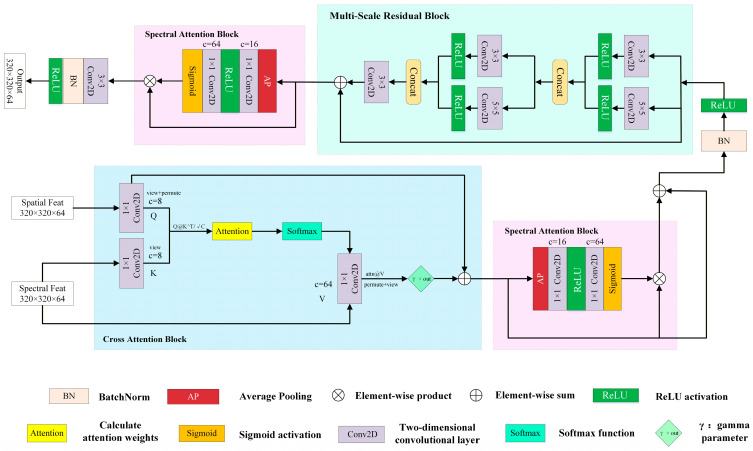
Detailed architecture of FusionNet. The diagram details the layer parameters. For layers where the stride is not specified, it defaults to 1, and the number of output channels defaults to 64.

Directly concatenating the feature maps extracted from the spatial stream and the spectral stream essentially reduces the network to a simple single-stream feature concatenation. Subsequent convolutional layers are required to process both spatial details and spectral information simultaneously, which can easily lead to interference between features of different modalities. To avoid this issue, we first introduced a cross-attention block [62] in the FusionNet (Figure 4), which dynamically adjusts feature weights by calculating the similarity matrix between the spatial and spectral stream feature maps. In this block, the spatial stream serves as the Query, while the upsampled spectral stream functions as the Key and Value, generating dynamic weights via a softmax function to optimize cross-modal interactions. At this stage, the spectral features have not yet been interfered with by spatial information. We first use the first-layer spectral attention block (SAB) [63] to highlight key feature channels, ensuring the full preservation of spectral features. After optimization by the first-layer spectral attention, the resulting feature vectors, which preserve the spatial structure and enhance the spectral representation, are input into the Multi-Scale Residual Block (MSRB) [64]. MSRB can perform multi-scale modeling of spatial structures based on these features through parallel convolutional kernels with different receptive fields, thereby further enhancing the model’s ability to capture spatial information at different scales. Subsequently, the feature vectors output by the MSRB are fed into the second-layer SAB. This block is designed to suppress the residual noise that may be generated by multi-scale spatial convolutions and to recalibrate the spectral features, thereby further enhancing the spectral fidelity of key bands for water quality monitoring.

#### 3.2.3. Reconstruction Network

**Figure 5:**
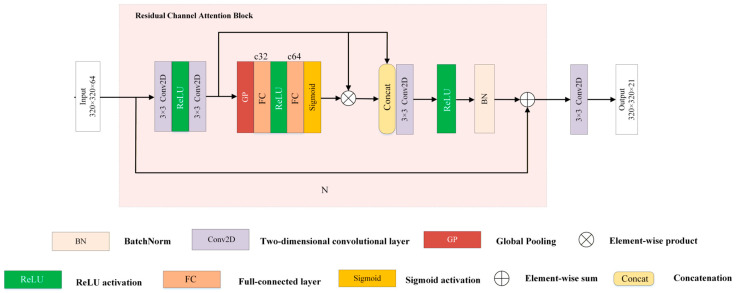
Detailed architecture of the RstNet. The diagram details the layer parameters. For layers where the stride is not specified, it defaults to 1, and the number of output channels defaults to 64.

RstNet (Figure 5) is responsible for remapping the fused feature vectors generated by FusionNet back into the image space to produce the final HRMS output. Its network architecture is similar to the spectral stream structure of EmbNet, incorporating a single RCAB to optimize the reconstruction process and employing a 3 × 3 convolutional layer to directly output the reconstructed high-resolution fusion result with 21 bands. Additionally, to maintain spectral consistency and training stability, a global skip connection is employed between the original low-resolution S3 OLCI images and the generated HRMS images.

### 3.3. Discriminative Network of MSA-UGAN

In the absence of authentic HRMS images, MSA-UGAN does not aim to generate images that resemble ideal labels. Instead, it ensures that the generated HRMS images maintain consistency with the spatial and spectral features of the input raw data through the constraints imposed by the network architecture and loss functions. Therefore, the discriminative network no longer functions as a traditional binary classifier distinguishing real images from generated ones, but rather guides the training of the generative network by assessing whether the spatial and spectral features of the generated images originate from real data characteristics.

In this study, the discriminative network employs the PatchGAN architecture [31] (additional details can be obtained in Figure 2). Unlike traditional methods that evaluate the entire image, PatchGAN assesses the authenticity of local regions of an image (i.e., image patches) through convolution operations. This design enables a more accurate capture of the spatial details and texture features of S2 MSI. Additionally, PatchGAN has a simple design. All convolutional layers use 4 × 4 convolutions, with a stride of 2 for the first three layers and a stride of 1 for the last two layers. The number of channels is 64, 128, 256, 512, and 1, respectively. Furthermore, each layer employs the LeakyReLU activation function, along with instance normalization to stabilize the training process and enhance network performance. These design choices make it well-suited for unsupervised image generation tasks. Therefore, in the spatial—spectral fusion task of S2 MSI and S3 OLCI, we adopt the PatchGAN architecture as *D*_1_.

### 3.4. Loss Function of MSA-UGAN

The hybrid loss function in this paper refers to the four loss terms of the original UCGAN [32], namely the cycle consistency loss (*L_cyc_*), adversarial loss (*L_adv_*), reconstruction loss (*L_rec_*), and no-reference quality loss (*L_nrf_*). To further constrain the spectral information of the fused image, inspired by the cycle consistency learning framework in UCGAN, we introduced a downsampling consistency strategy into the spectral domain and designed a spectral angle loss based on downsampling consistency (*L_DCSA_*) to directly constrain the spectral fidelity of the fusion result. The overall objective function can be expressed as

*L*_*G*1_ = λ_1_*L_rec_* + λ_2_*L_adv_* + λ_3_*L_cyc_* + λ_4_*L_DCSA_* + λ_5_*L_nrf_* (1)

where λ_1_, λ_2_, λ_3_, λ_4_, and λ_5_ are the weight coefficients that balance the contributions of the individual loss terms, and they are set to λ_1_ = 5 × 10^−4^, λ_2_ = 1 × 10^−3^, λ_3_ = 1 × 10^−3^, λ_4_ = 1, and λ_5_ = 1. Regarding the determination of weights, since the proposed model is optimized based on UCGAN, we continue to adopt the weight parameters of the four loss terms from UCGAN. Furthermore, we conducted a sensitivity analysis on the *L_DCSA_* weight to ascertain its final value. Next, we will introduce them in order.

#### 3.4.1. Reconstruction Loss

In an unsupervised setting, due to the absence of true labels, *L_rec_* is introduced to constrain the generation process and ensure that the input information is preserved. Accordingly, *L_rec_* is set to impose constraints in the S2_S3_SRF_ and S3 OLCI domains, thereby achieving better spatial and spectral preservation. S2_S3_SRF_ and S3 OLCI are reconstructed from the spatial and spectral fused output, and then we can evaluate the consistency with these two original inputs. Finally, the total *L_rec_* is a sum of spatial reconstruction loss (*L_spatial_*) and spectral reconstruction loss (*L_spectral_*).

*L_rec_* = *L_spatial_* + *L_spectral_* (2)

For *L_spatial_*, we focus on high-pass features extracted from S2_S3_SRF_, while *L_spectral_* emphasizes low-pass components derived from S3 OLCI.

*L_spatial_* = ‖ *gethp* (*Y*) − *MP* (*gethp* (*G*_1_ (*X*, *Y*))) ‖_1_ (3)

where *X* and *Y* represent the input S3 OLCI and S2_S3_SRF_ images, respectively. *G*_1_ is the dual-stream generative network, *gethp* (·) extracts the high-pass information from the image, *MP* (·) is the maximum pooling operation for keeping the spatial details, and ‖·‖_1_ is the *Ɩ*_1_ norm.

*L_spectral_* = ‖ *getlp* (*X*) − *getlp* (*G*_1_ (*X*, *Y*) ↓) ‖_1_ (4)

where *getlp* (·) extracts the low-pass information from the image, and ↓ means the down-sample operator which degrades the fused result back to the S3 OLCI resolution. In this paper, bicubic interpolation is consistently employed for spatial dimensionality reduction in images. Low-pass information is acquired by applying an average filter to the original image, whereas high-pass information is obtained by subtracting the low-pass information, the result of the average filter, from the original image.

#### 3.4.2. Adversarial Loss

In the absence of authentic HRMS reference images, we employ a GAN architecture for unsupervised learning. However, since the training data is typically acquired through synthetic degradation, the model optimized in this dynamic adversarial training may exhibit performance discrepancies when applied to full-resolution real images [65]. Therefore, the *L_adv_* [32] based on the cycle consistency mechanism is set to address the issue of missing reference images in unsupervised scenarios and to enhance the adaptability of MSA-UGAN to real data. The *L_adv_* term on *G*_1_ can be formulated as

*L_adv-G_*_1_ = (*D*_1_ (*Y*, *G*_1_ (*X*, *Y*) ↓, *G*_1_ (*G*_1_ (*X*, *Y*) ↓, *Y*)) − 1)^2^ (5)

where *X* and *Y* represent the input S3 OLCI and S2_S3_SRF_ images, respectively. *G*_1_ is the dual-stream generative network, *D*_1_ stands for the discriminative network, ↓ means the down-sample operator which degrades the fused result back to S3 OLCI resolution. The purpose of downsampling the spectral information of the generated HRMS images is to obtain an image with spectra consistent with the original S3 OLCI data. The downsampled result will be used as a pseudo-input and fed back into *G*_1_. By comparing the differences between the two generated HRMS images, unsupervised training can be achieved without the need for real data labels.

Similarly, we can formulate the *L_adv_* for *D*_1_

*L_adv-D_*_1_ = (*D*_1_ (*Y*, *X*, *G*_1_ (*X*, *Y*)) − *a*)^2^ + (*D*_1_ (*Y*, *G*_1_ (*X*, *Y*) ↓, *G*_1_ (*G*_1_ (*X*, *Y*) ↓, Y)) − *b*)^2^ (6)

where *a* and *b* are the soft labels near 1 and 0 respectively to avoid the over-fitting in *D*_1_.

#### 3.4.3. Cycle-Consistency Loss

*L_cyc_* [66] is set to ensure the consistency of fusion results during scale transformation without relying on reference images. First, the generated HRMS images are downsampled back to the original S3 OLCI scale and used as pseudo-inputs to re-enter *G*_1_, reconstructing the HRMS. By minimizing the difference between the originally generated and reconstructed results, self-supervised constraints on spectral fidelity and structural stability are achieved without the need for real data labels. This loss term can be formulated as

*L_cyc_* = ‖ *G*_1_ (*X*, *Y*) − *G*_1_ (*G*_1_ (*X*, *Y*)↓, *Y*) ‖_1_ (7)

where *X* and *Y* represent the input S3 OLCI and S2_S3_SRF_ images, respectively. *G*_1_ is the dual-stream generative network, ↓ means the down-sample operator which degrades the fused result back to S3 OLCI resolution.

#### 3.4.4. Downsampling-Consistent Spectral Angle Loss

The Spectral Angle Mapper (SAM) is one of the commonly used criteria to assess the fused images. Since SAM denotes the absolute value of the spectral angle between the two feature maps, it can provide information about the degree of spectral distortion irrespective of their illumination conditions [67], which is crucial for lake water quality monitoring tasks. Lake water typically contains various suspended solids, chlorophyll, and colored dissolved organic matter, making its optical properties more complex than those of cleaner water bodies such as seawater. Therefore, in the context of lake water quality monitoring, the requirements for preserving spectral fidelity in fused images are more stringent. Although *L_cyc_* can indirectly maintain the spectral structure of the original low-resolution images, its core function is to ensure the consistency of the fusion results during scale transformation. It does not directly optimize the accuracy of spectral shapes and may produce images where pixel values match but the spectral shapes are distorted, thereby affecting the accuracy of water quality monitoring tasks and the scientific validity of subsequent decision-making. To this end, we designed a spectral angle loss function based on downsampling consistency (*L_DCSA_*) as a constraint for spectral enhancement [32,68]. In the absence of real HRMS data, we used the input low-resolution S3 OLCI image *X* as the spectral reference. Specifically, the high-resolution fusion result *G*_1_(*X*, *Y*) output by the generative network is downsampled to the same resolution as the S3 OLCI image using bicubic interpolation, resulting in *G*_1_(*X,Y*)↓. We then calculate the spectral angle between *G*_1_(*X,Y*)↓ and *X* on a pixel-by-pixel basis. The design of this loss function is based on the following unsupervised principle: an ideal high-resolution fused result, when downscaled to low resolution, should retain spectral features consistent with the original low-resolution observed image. Therefore, this loss term can directly constrain the spectral features of the fused image to remain consistent with the original S3 OLCI. It can be calculated as

(8) $$L_{\mathrm{DCSA}}=\frac{1}{H\times W}\sum_{i=1}^{H}\sum_{j=1}^{W}\cos^{-1}(\frac{\langle {(G}_{1}\;(X,\;Y)↓{)}_{i,j},\;X_{i,j}\rangle }{\|\;(G_{1}\;(X,\;Y)\;{{↓)}_{i,j}\;\|}_{2}\;\cdot \;\|\;X_{i,j}{\|}_{2}+\varepsilon })$$

where *X* and *Y* represent the input S3 OLCI and S2_S3_SRF_ images, respectively, *G*_1_ is a dual-stream generative network, ↓ means the down-sample operator which degrades the fused result back to S3 OLCI resolution. By calculating the spectral angle between the downsampled fused result and the original S3 OLCI image, it ensures that the spectral information of the fused result remains consistent with the input S3 OLCI image, thereby preventing spectral distortion. ‖·‖_2_ is the *Ɩ*_2_ norm, and ε is a minimum value to prevent division by zero errors.

#### 3.4.5. No Reference Loss

Relying solely on *L_adv_* and *L_rec_* within an unsupervised framework may not adequately constrain the spatial and spectral fidelity of fused images. Therefore, we used the *L_nrf_* to further improve the overall spatial and spectral quality of the fused images. The *L_nrf_* is calculated as

*L_nrf_* = 1 − *QNR* (9)

where QNR is a no-reference image quality assessment metric, and QNR = (1 − D_λ_)(1 − D_S_). The QNR calculation in our implementation follows the formulation in [56] and is implemented using PyTorch (version 2.0.1) with Python 3.11.13. Specifically, the universal image quality index used in D_λ_ and D_S_ calculations includes a small constant ε = 1 × 10^−8^ in the denominator to ensure numerical stability and prevent division by zero. While the absolute value operations in the original QNR formulation are mathematically non-differentiable at zero, the `torch.abs () ` function in PyTorch defines a subgradient of 0 at this point, which allows gradient-based optimization. This approach of using QNR as a differentiable loss term has been proven effective in UCGAN [32].

#### 3.4.6. Ablation Study for *L_DCSA_*

In this paper, we refer to four loss terms for UCGAN and add a spectral angular loss term based on downsampling consistency (*L_DCSA_*). To verify the impact and performance of this loss function, we compared the full five losses with the four losses without the *L_DCSA_*. All variables are trained and tested under the same scheme, except for the loss function. Table 2 shows the results of the ablation study conducted on the designed dataset. When the *L_DCSA_* was introduced into our model, the QNR increased, and the D_λ_ decreased from 0.0327 to 0.0252, indicating an improvement in the overall quality of the fusion results and a reduction in spectral distortion. At the same time, the D_S_ did not change significantly, which indicates that after the introduction of the *L_DCSA_*, the model did not have a significant negative impact on the preservation of spatial details while ensuring the improvement of spectral fidelity.

In addition, we also discussed the hyperparameter settings of *L_DCSA_* (Table 3). For the weight variable of *L_DCSA_*, we conducted experiments with three different values. The test results under different parameter settings indicate that when the weight variable is set to 1, the overall fusion performance is optimal, with QNR reaching its maximum and D_λ_ decreasing to its minimum. When the weight variable increases to 10, QNR slightly decreases, D_λ_ remains almost unchanged, and D_S_ shows a slight increase. This may be due to the excessive weight of the *L_DCSA_*, causing the model to focus too much on spectral consistency at the expense of spatial information fusion accuracy to some extent. When the weight variable is reduced to 0.1, QNR drops to its lowest level, D_λ_ rises to its highest level, and D_S_ remains nearly unchanged. This indicates that the constraint strength of *L_DCSA_* is insufficient in this case, failing to fully leverage its ability to enhance spectral fidelity. Therefore, we set the weight variable of *L_DCSA_* to 1 as the final parameter setting.

### 3.5. Training Details

All experiments were conducted on a single NVIDIA RTX 4070 GPU, implemented using PyTorch (version 2.0.1) with Python 3.11.13. The total number of trainable parameters is 1,305,382, comprising 589,765 parameters in *G*_1_ and 715,617 parameters in *D*_1_. The model was trained for 10,000 iterations with a batch size of 4, and the total training time was approximately 2.9 h. Both *G*_1_ and *D*_1_ were trained by the AdamW optimizer. with initial learning rates set to 1 × 10^−4^ and 5 × 10^−5^ respectively. Considering the balance between convergence speed and stability, along with the configuration of training parameters, the learning rates were multiplicatively decayed by a factor of 0.9 every 2000 iterations. The soft label *a* is a random number ranging from 0.7 to 1.2 and the soft label *b* is a random number ranging from 0 to 0.3. During training, the peak GPU memory consumption was approximately 4.2 GB. In the testing phase, the inference time for processing a single set of input images was 0.0067 s.

## 4. Results

In this section, we demonstrate the performance of the proposed MSA-UGAN on S2_S3_SRF_ and S3 OLCI datasets (described in Section 2.2) and compare it with several benchmark algorithms. It should be noted that the comparative experiments in this study aim to verify the feasibility of the proposed Unsupervised GAN-based method for the spatial–spectral fusion task of heterogeneous sensors, S2 MSI and S3 OLCI, rather than pursuing a comprehensive performance comparison with all state-of-the-art models. Therefore, the selection of comparative models focuses on three traditional algorithms: GS [13], SFIM [18], and MTF_GLP [19], as well as two DL-based algorithms: the supervised SRCNN [69,70] and the unsupervised UCGAN [32]. This selection encompasses a diverse array of mainstream algorithms from different technical pathways. The codes of all these compared methods are publicly accessible, and we utilize the same dataset partitioning and evaluation metrics as in this study.

Specifically, in DL-based models, SRCNN was extended into a dual-input network as a comparative model for the supervised method. During the training phase, the S2_S3_SRF_ images (60 m) are downsampled to 300 m by a factor of 5 using bicubic interpolation as spatial auxiliary input, while the corresponding real S3 OLCI images (300 m) serve as the primary spectral input. The supervision target is the 60 m S2_S3_SRF_ image. In the testing phase, the real S3 OLCI images (300 m) and the 300 m S2_S3_SRF_ images obtained through bicubic downsampling at the same time are jointly input into the trained SRCNN model to output the 60 m fused image. For UCGAN, the experimental settings for training and testing were consistent with those of MSA-UGAN. All DL-based models were trained until the loss stabilized, ensuring comparisons were made under convergence conditions.

For non-reference metrics, namely D_λ_, D_S_, and QNR, the experiments were conducted on full-resolution images using S2_S3_SRF_ and S3 OLCI images as inputs. For reference metrics, namely SAM, ERGAS, and SSIM, the experiments followed the Wald protocol [71], where S2_S3_SRF_ was downsampled to 300 m resolution and then input into the model together with S3 OLCI images, while the 60 m resolution S2_S3_SRF_ image served as the reference HRMS image. This setup is intended to ensure the fairness of comparisons among methods, so as to demonstrate the performance limits that each method can achieve under ideal conditions.

### 4.1. Qualitative Analysis

**Figure 6:**
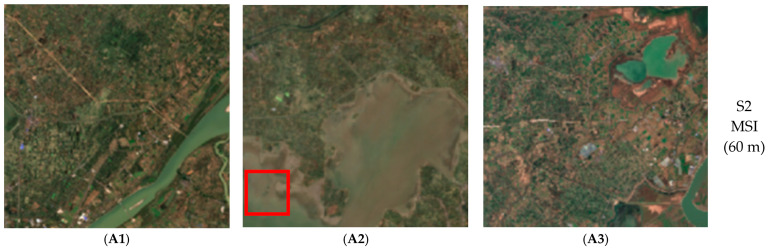

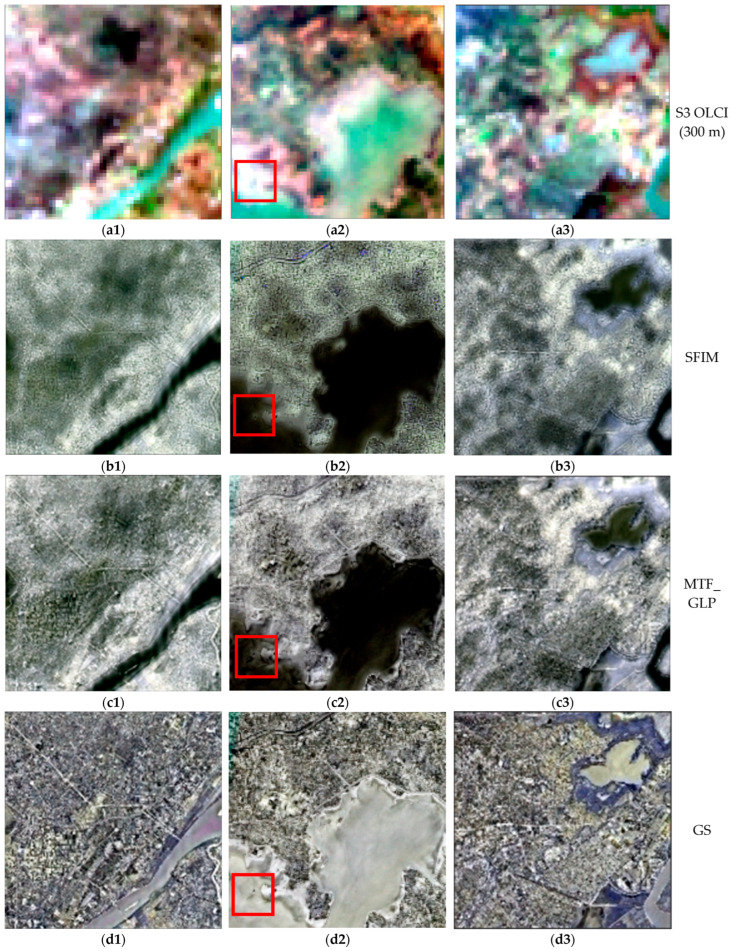

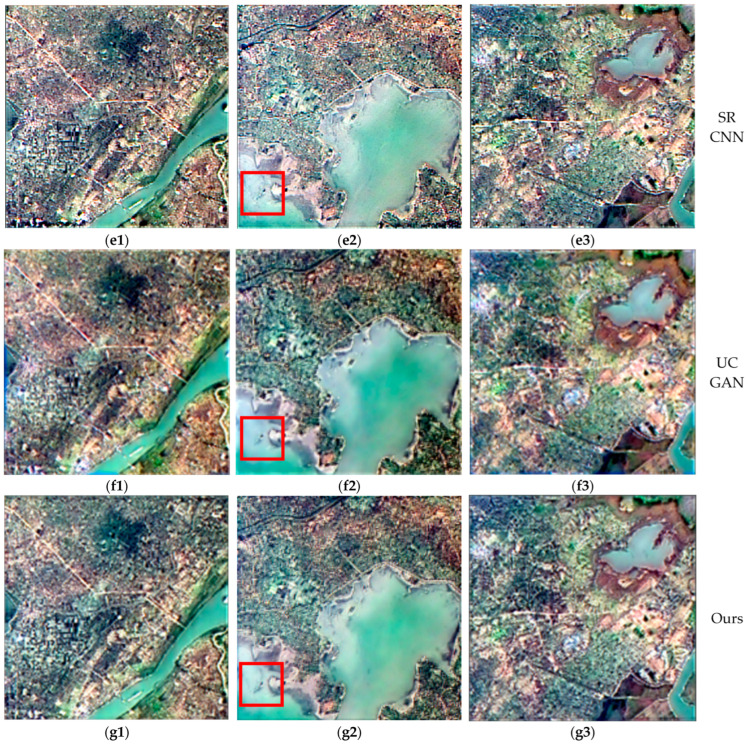
Qualitative results of the comparative experiments. Visualized in RGB. The red box highlights a local region used for detailed texture comparison. (**A1**–**A3**) S2 MSI. (**a1**–**a3**) S3 OLCI. (**b1**–**b3**) SFIM. (**c1**–**c3**) MTF_GLP. (**d1**–**d3**) GS. (**e1**–**e3**) SRCNN. (**f1**–**f3**) UCGAN. (**g1**–**g3**) ours.

Figure 6 presents the qualitative results of six methods. Visual analysis clearly indicates that, overall, the fused images generated by MSA-UGAN (g1–g3) have the highest overall quality when compared with the results of the three traditional methods (b1–b3, c1–c3, d1–d3, corresponding to SFIM, MTF_GLP, and GS, respectively), the results of two DL-based methods (e1–e3, f1–f3, corresponding to SRCNN and UCGAN, respectively), and the original S3 OLCI images (a1–a3).

Overall, MSA-UGAN exhibits reduced color distortion and fewer halo effects compared to other methods, while offering improved clarity in textures and edges. Small water bodies such as lakes and rivers, which were relatively blurry at 300 m resolution, are now clearer in the 60 m fused images. In contrast, compared to the original S3 OLCI images, the GS method enhances the boundaries and texture details of the fused images but suffers from significant color distortion. SFIM and MTF_GLP introduce varying degrees of noise and artifacts, leading to blurred boundaries, a noticeable loss of detail, and pronounced color distortion. Although the fused images generated by SRCNN and UCGAN outperform traditional methods in terms of texture and edge clarity, the edge quality of SRCNN images is lower compared to MSA-UGAN, while UCGAN images exhibit some halo effects and color distortions at the edges. Furthermore, both methods show some loss of spatial details for features such as rivers.

Locally, taking the red-boxed area in Figure 6 as an example, the texture details of the lake are difficult to distinguish in the S3 OLCI image. The visual effect in the red-boxed area of SFIM and MTF_GLP is the most affected, introducing numerous artifacts and rendering the texture details poorly defined. The fusion result of GS in the red-boxed area restores the texture details better than SFIM and MTF_GLP, but exhibits color distortion and some reduction in overall clarity. Although the overall quality of the fusion results of UCGAN and SRCNN is visually improved, the texture details of the lakes in the red-framed area are somewhat missing to a certain extent, indicating that the two models do not fully preserve fine-scale texture details. In contrast, MSA-UGAN better preserves the texture details of the lakes in the red-boxed areas, with its texture features being clear and complete. This demonstrates that MSA-UGAN can better restore the high-frequency texture features of geographic elements.

### 4.2. Quantitative Analysis

To further validate the performance of the proposed MSA-UGAN, we complement the qualitative observations with a quantitative evaluation, as summarized in Table 4. For the complete images, the proposed MSA-UGAN achieves D_λ_ = 0.0252, D_S_ = 0.0389, and QNR = 0.9709, outperforming the compared methods across all three metrics. Specifically, compared to UCGAN, MSA-UGAN reduces D_λ_ from 0.0389 to 0.0252 and D_S_ from 0.0452 to 0.0389, with QNR increasing from 0.9358 to 0.9709. Compared with SRCNN, D_λ_ decreases from 0.0453 to 0.0252, D_S_ from 0.0475 to 0.0389, and QNR improves from 0.9227 to 0.9709. It should be noted that although the numerical changes in the evaluation metrics are relatively small, we have visually presented their range of uncertainty by reporting the mean ± standard deviation of each metric (Table 4). Therefore, this improvement remains reliable within the framework of statistical testing.

Among traditional approaches, GS achieves moderate spectral preservation (D_λ_ = 0.0429) but exhibits the highest spatial distortion (D_S_ = 0.2668) among all methods, resulting in a QNR of 0.7037. MTF_GLP shows the highest spectral distortion (D_λ_ = 0.0926) combined with substantial spatial distortion (D_S_ = 0.1697), leading to a QNR of 0.7560. SFIM demonstrates improved spatial quality (D_S_ = 0.1468) and lower spectral distortion (D_λ_ = 0.0740) compared to MTF_GLP, yet its QNR remains at 0.7921. While traditional methods such as GS, SFIM, and MTF_GLP have proven effective in specific fusion scenarios (e.g., pan-sharpening or homogeneous sensor data fusion), their performance in cross-sensor fusion tasks involving S2 MSI and S3 OLCI is limited. This limitation can be attributed to their reliance on simplified mathematical transformations or linear component substitution, which have difficulty in modeling the complex nonlinear relationships inherent in multi-source remote sensing data. As a result, these methods encounter difficulties in simultaneously preserving spectral fidelity and spatial details when applied to the heterogeneous sensor pair considered in this study.

Overall, as can be seen from Table 4, MSA-UGAN achieves relatively the best results on full-size images and also obtains satisfactory results on reference metrics, indicating its practical value in real-world applications.

### 4.3. Spectral Fidelity Assessment of Fused Images via Spatial Downsampling

To further assess the spectral fidelity of the fused images in relation to the original S3 OLCI data, we downsampled the fused images to a resolution of 300 m to mimic the spatial resolution of the original S3 OLCI data. Subsequently, we extracted the spectral reflectance data from the downsampled fused images and the original S3 OLCI images at corresponding pixels for spectral analysis. The types of the extracted pixels were water bodies. Quantitative evaluation was carried out based on the indices of the root mean square error (RMSE↓) and the correlation coefficient (CC↑).

**Figure 7:**
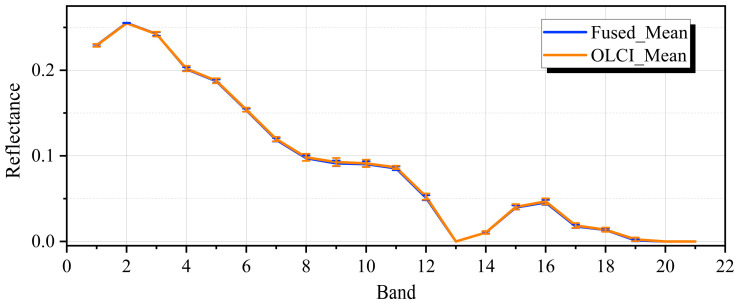
The mean spectral curve of water body areas in S3 OLCI and fused image. The vertical lines at each band indicate the standard deviation.

Figure 7 displayed the mean spectral curve and standard deviation of 21 bands over water bodies for the fused image and the original S3 OLCI image. It is seen that the mean spectral curve of the fused image closely aligns with that of the original S3 OLCI image, and the spectral shape and variation trend remain consistent across the entire spectral range. This indicated that our fusion model largely preserved the original spectral characteristics of S3 OLCI. Meanwhile, the standard deviations for each band were relatively small, suggesting that the fused image demonstrated strong spectral stability across different pixels without introducing excessive spectral noise.

For quantitative evaluation, the accuracies of water bodies are presented in Table 5. It can be observed that the CC values of all experimental pixels are relatively high, with an average of 0.9998 and a minimum of 0.9991. At the same time, the RMSE values are relatively low, averaging 0.0250 and reaching a maximum of 0.0429. This further indicated that the fused data exhibit high spectral consistency with the S3 OLCI data in water body regions and that our fusion model effectively preserved the spectral characteristics of the original S3 OLCI images.

It should be noted that this validation method implicitly assumes that downsampling can faithfully preserve spectral information. This comparison mainly evaluates the spectral consistency of the fusion results at the original observation scale. Although it cannot completely reveal potential sub-pixel nonlinear distortions, due to the difficulty of acquiring true HRMS images, this method, as one of the commonly used approaches in the field of remote sensing fusion for verifying spectral fidelity [71], can effectively assess the radiometric consistency of the fusion results at the original low-resolution sensor scale.

## 5. Chl-a Concentration Inversion

### 5.1. Data Sources for Chl-a Concentration Inversion

**Figure 8:**
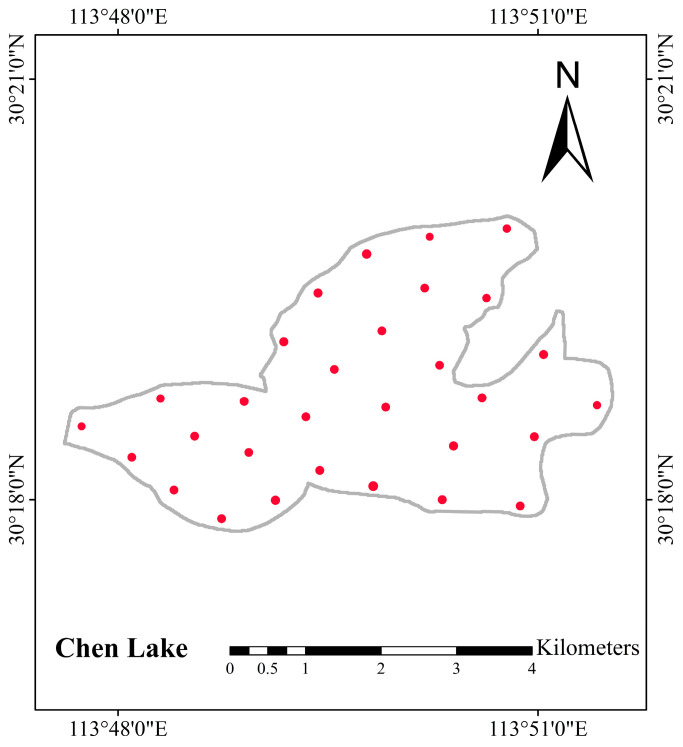
Spatial Distribution Diagram of Sampling Points of Chen Lake. The gray lines represent the range of the Chen Lake, and the red dots represent the sampling points.

This study collected 30 water quality samples from Chen Lake on 18 April 2025, distributed across different areas of the lake, covering both the central and coastal regions, with uniform spatial coverage. The sampling was conducted on a cloud-free day with clear skies and good visibility (Figure 8). At each sampling point, water samples were collected 0.5 m below the surface using an Organic Glass Water Sampler, and the latitude and longitude coordinates of the sampling points were recorded on-site with a handheld GPS device. The data on Chl-a concentration, along with the latitude and longitude coordinates of each sampling point, were collected, submitted for testing, and organized before provided by the Landscaping Bureau of Caidian District, Wuhan City, Hubei Province. Statistical analysis indicated that the Chl-a concentration across the 30 sampling sites ranged from 3.47 to 18.19 µg/L, with a mean value of 8.21 µg/L. The satellite overpass coincided with the sampling date. Consequently, remote sensing data were obtained from a cloud-free S2 MSI L2A and S3 OLCI L1B images acquired on 18 April 2025, which was downloaded from the ESA’s Copernicus Open Access Hub (https://browser.dataspace.copernicus.eu/, accessed on 29 September 2025).

To obtained the fused image for chl-a inversion, we first cropped the S2 MSI and S3 OLCI images to the same area of Chen Lake. The detailed preprocessing procedure is described in Section 2.2. Subsequently, HRMS image for the corresponding period was generated using MSA-UGAN, and the water spectral reflectance data at each sampling point in Chen Lake were extracted from the fused image. For the S2 MSI images used in collaborative inversion, we first resampled bands B8 and B10 in the S2 MSI files to a 60 m resolution using bicubic interpolation. This is because the originally downloaded S2 MSI L2A files already provide surface reflectance products at 60 m resolution for the remaining 11 bands. Subsequently, the 13 bands of S2 MSI data at 60 m resolution were combined into a multispectral image to facilitate subsequent processing. Finally, the downsampled image was clipped to the Chen Lake area and the spectral reflectance data of water bodies at each sampling point were extracted from this image.

### 5.2. Correlation Analysis Between Chl-a Concentration and Spectral Bands

To select spectral bands sensitive to Chl-a concentration from the S2 MSI image and the fused image, we employed Pearson correlation analysis to quantify their relationship with in situ measurements, thereby determining the optimal inputs for subsequent machine learning algorithms. The correlation coefficient *r* ranges from [−1, 1], where positive values indicate positive correlation and negative values indicate negative correlation. A higher value signifies stronger correlation between the variables.

Using this approach, we screened single bands that are sensitive to Chl-a concentration. Based on these sensitive single bands, various band combinations were constructed, including band differences, band ratios, and normalized difference indices. These combinations aim to enhance the spectral signal related to Chl-a while reducing the influence of water constituents. The results of the correlations are presented in Table 6.

As shown in Table 6, the measured Chl-a concentrations are significantly positively correlated with the Oa11 of S3 OLCI data, and significantly negatively correlated with the Oa3 and Oa8. Moreover, the combination of spectral bands markedly enhances the correlation. Notably, the normalized difference index (Oa11 − Oa8)/(Oa11 + Oa8) exhibited a correlation coefficient of up to 0.85 with Chl-a concentration, which demonstrates the effectiveness of red-edge and red band combinations for Chl-a estimation [72,73,74]. This is likely because the index, by comparing the near-infrared reflectance peak with the red-light absorption peak, effectively enhances the spectral response of Chl-a while reducing interference from factors such as suspended matter and colored dissolved organic matter, thereby demonstrating high sensitivity to variations in Chl-a concentration. Additionally, the ratios Oa6/Oa8 and Oa11/Oa8 also exhibit strong correlations (r > 0.75).

For the S2 MSI data from synergistic inversion, B1, B3, and B5 exhibit a high correlation with the measured Chl-a concentrations. In particular, the normalized difference chlorophyll index, (B5 − B4)/(B5 + B4), has a correlation coefficient as high as 0.81. This is mainly due to the index’s strong absorption of Chl-a in the red band and its high reflectance in the near-infrared band, effectively highlighting the spectral response characteristics of Chl-a [75,76]. Additionally, the band ratios B5/B4 and B5/B1 also show a high correlation with the measured Chl-a concentrations (r > 0.70).

By comprehensively testing all possible band combinations, while considering the high collinearity among variables and the potential impact of small-sample datasets on the models, the following indicators were ultimately selected as input parameters for the Chl-a concentration inversion models. For S3 OLCI data, Oa3 and (Oa11 − Oa8)/(Oa11 + Oa8) were utilized, and for S2 MSI data, B1, B3, and (B5 − B4)/(B5 + B4) were selected. These sensitive bands and band combinations were served as input variables for the machine learning models, while the output variable was the predicted Chl-a concentrations.

### 5.3. Chl-a Concentration Inversion Model

#### 5.3.1. Selection of Machine Learning Models

In this study, three machine learning algorithms—SVR, RF, and XGBoost—were employed to develop models for inverting Chl-a concentration in Chen Lake. The selection was based on the advantages of each model in tackling the challenges posed by a limited sample size (n = 30) and in making the output predictions of Chl-a concentration more reliable. Specifically, SVR employs kernel functions to manage both linear and nonlinear relationships by mapping input variables into higher-dimensional spaces, an approach which enables good performance even with limited sample data [77]. RF was selected for water quality inversion due to its proven robustness in handling high-dimensional, non-linear relationships with limited samples. Its broad utility in extracting key features from complex, structured datasets has been demonstrated across diverse fields, including predictive analytics in virtual environments [78]. The mechanism operates by constructing numerous decision trees through the random selection of subsets of samples and features from the training data. The overall prediction is then derived by averaging the outputs of all the decision trees. This ensemble strategy can effectively reduce the risk of overfitting and enhance generalization ability, thus rendering the output predictions of Chl-a concentration more reliable. Similarly, XGBoost builds upon decision trees iteratively, with each new tree aiming to correct the residual errors of its predecessor. This process allows the predicted values to gradually converge towards the true values, making it more robust in small—sample scenarios where traditional algorithms might falter.

#### 5.3.2. Hyper-Parameter Optimization

To obtain robust hyperparameter combinations under small sample conditions (n = 30) and to avoid overfitting, we employed 5-fold cross-validation combined with external random search for hyperparameter optimization, using the cross-validated R^2^ and RMSE as optimization metrics. Specifically, the hyperparameter search spaces for SVR and RF were defined based on the implementations in the Scikit-learn library, whereas those for XGBoost were defined according to the XGBoost library. First, random search was conducted within a predefined hyperparameter space, generating 100 sets of hyperparameter combinations. Subsequently, the original dataset was first randomly divided into five mutually exclusive subsets, each containing six samples, ensuring consistency in data distribution across subsets. In each cross-validation iteration, four subsets (a total of 24 samples) were used as the training set, while the remaining subset (6 samples) served as the validation set. For each hyperparameter combination, the model is trained using the training dataset, and the cross-validated R^2^ and RMSE are computed on the validation set. After performing 5-fold cross-validation, the R^2^ and RMSE for each hyperparameter set are averaged across the five validation sets. The hyperparameter combination with the highest average R^2^ and the lowest average RMSE is chosen as the optimal set. The optimal hyperparameters for each machine learning algorithm, verified through repeated experiments using this process, are detailed in Table 7.

### 5.4. Superior Performance of RF Beyond SVR and XGBoost

Table 8 presents the predictive performance of three Chl-a inversion models. Among the models built using reflectance data from the fused image, the RF demonstrated relatively better performance (R^2^ = 0.87, RMSE = 3.17 µg/L, MAE = 2.29 µg/L), outperforming both SVR (R^2^ = 0.76, RMSE = 3.67 µg/L, MAE = 2.51 µg/L) and XGBoost (R^2^ = 0.85, RMSE = 3.24 µg/L, MAE = 2.35 µg/L). In the models constructed using S2 MSI image reflectance data, the RF still performed relatively better (R^2^ = 0.75, RMSE = 3.70 µg/L, MAE = 2.51 µg/L), outperforming SVR (R^2^ = 0.63, RMSE = 4.22 µg/L, MAE = 2.93 µg/L) and XGBoost (R^2^ = 0.72, RMSE = 3.76 µg/L, MAE = 2.54 µg/L).

This is likely attributable to the fact that the RF algorithm is an ensemble method based on regression decision trees. By combining multiple regression tree models into a model ensemble, it enhances generalization ability and improves model accuracy. Meanwhile, compared to boosting methods or single-kernel models, ensemble strategies such as bagging are more robust to noise and small sample sizes [79,80]. Specifically, since the dataset used in the experiment is relatively small, these models are more likely to be disrupted by random noise during training. Furthermore, RF’s bagging mechanism, which builds multiple trees and averages their predictions, is known to reduce variance and mitigate overfitting, making it well-suited for the small-sample learning scenario in this study. In contrast, XGBoost, as a boosting-based method, sequentially fits residuals and tends to be more sensitive to noise in small datasets, which can lead to overfitting despite its built-in regularization. As for SVR, its performance depends on the choice of kernel function and hyperparameter settings. In high-dimensional feature spaces with limited samples, it often has difficulty capturing complex patterns and may underfit or become unstable [81]. Therefore, in this study, the advantage of the RF algorithm is jointly determined by its algorithmic characteristics and the characteristics of the dataset used in this study.

Overall, models using reflectance data from the fused image show higher accuracy than those using reflectance data from S2 MSI. This indicates that the fused image can provide richer and more detailed spectral information, thereby enhancing the accuracy of Chl-a inversion. Therefore, this study selected the RF to generate a spatial distribution map of Chl-a concentration across the entire lake.

### 5.5. Spatial Distribution of Chl-a Concentration in Chen Lake

**Figure 9:**
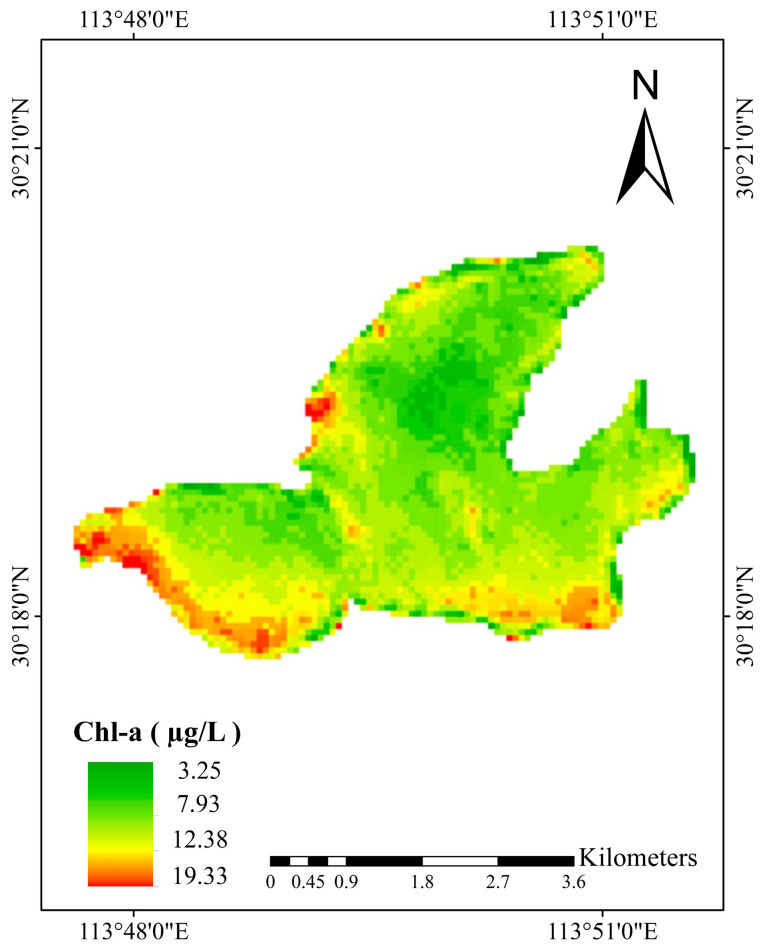
Spatial distribution of Chl-a concentration in Chen Lake on 18 April 2025.

The preliminary spatial distribution based on the RF model is shown in Figure 9. Owing to the limited sample size (n = 30), this study presents the distribution merely as the results of a preliminary exploratory analysis. It is shown that the total Chl-a concentration in Chen Lake predominantly ranged from 3.25 to 19.33 µg/L. According to the Comprehensive Trophic Level Index (TLI) in the ‘Environmental Quality Standards for Surface Water’ (GB 3838-2002) [82] issued by the Ministry of Ecology and Environment, the eutrophication assessment standard using Chl-a as a single indicator indicates that this concentration range reflects the overall water quality of Chen Lake in a transitional state between mesotrophic and mildly eutrophic. Spatially, high Chl-a concentrations were primarily observed in the southern part of the lake, especially in the southwestern nearshore area, whereas lower values were recorded in the central and northeastern regions. This spatial heterogeneity suggests a potential link to land use patterns. The southern watershed of Chen Lake hosts extensive aquaculture and crop cultivation activities. These activities are potential sources of exogenous nutrients, as aquaculture can release residual feed and waste, and agricultural runoff may carry fertilizer residues into the lake through surface runoff or groundwater infiltration. Such nutrient loads may create favorable conditions for algal blooms in the nearshore areas of the southern region. However, due to the lack of relevant literature on Chen Lake sediments and the absence of field data on nutrient concentrations and hydrological parameters, the above analysis can be regarded as a reasonable exploration of the potential causes for the increased Chl-a concentrations in this area.

In response to these environmental challenges, the Caidian District government has implemented a series of restoration initiatives in the Chen Lake Wetland Nature Reserve in recent years. Key measures include the removal of enclosed aquaculture nets, fishing nets, and bird nets (collectively known as the “three nets”), the establishment of domestic sewage treatment systems around the wetland, and the implementation of the practice of returning farmland to lake and wetland. However, on 18 April 2025, the relatively high concentrations of Chl-a exhibited in the southwestern area of Chen Lake indicated that this region is more likely to undergo eutrophication in the future. This also suggests that the ecological restoration process of the Chen Lake wetland has entered a critical stage, shifting from ‘overall improvement’ to ‘localized intensification,’ necessitating the scientific formulation of more precise management strategies.

## 6. Discussion

Although the proposed model effectively achieves spatial and spectral fusion of S2 MSI and S3 OLCI images, there remains room for improvement in the spatial resolution of the fused products. Secondly, the existing in situ Chl-a samples are only from Chen Lake and are limited in number, with the optical properties of this water body being relatively constant. When the Chl-a inversion model is directly applied to other water bodies with significantly different optical properties (such as clear deep lakes, highly turbid rivers, or nearshore sea areas), its fusion performance and inversion accuracy may be uncertain. This could, to some extent, constrain the generalization ability of the inversion model to other types of water bodies. Furthermore, although this study applied atmospheric correction to all input images, the atmospheric correction algorithm itself contains inherent uncertainties, particularly in cases of high aerosol optical thickness or where water bodies are adjacent to complex land features. These residual atmospheric correction errors may be amplified by the fusion model, thereby affecting the spectral fidelity of the fusion results.

Therefore, future research will proceed in the following directions: First, we will investigate the potential enhancement of the existing model using a stacked GAN [83]. Specifically, based on the current 60 m fusion results, we will attempt to introduce the 10 m bands of S2 MSI as spatial detail priors to gradually enhance the spatial resolution of the fused images. At the same time, we will introduce images from more regions and different time phases to construct multiple datasets, thereby validating the generalization capability of the optimized model under various geographical environments and seasonal conditions. Secondly, we will collaborate with more relevant environmental protection departments and research institutions to simultaneously collect hydrological data, including water depth, wind speed, water temperature, and nutrient concentrations. Additionally, we will obtain additional long-term observational data on Chl-a concentrations from various regions and types of water bodies to improve the generalization ability of the inversion model.

Meanwhile, in the future, this study will extend the application of the fused image technology generated by MSA-UGAN to the remote sensing inversion of other water quality parameters, such as suspended solids concentration, colored dissolved organic matter, and water transparency. It aims to establish a multi-parameter collaborative eutrophication assessment model to achieve a more comprehensive and in-depth evaluation of lake eutrophication phenomena. Additionally, it intends to explore expansion into broader environmental remote sensing tasks, such as harmful algal bloom dynamic monitoring and nearshore ecological environment change analysis. Furthermore, this study will systematically assess the adaptability of the MSA-UGAN under various atmospheric correction conditions in the future, aiming to verify its reliability and stability in practical applications, thereby providing technical support for subsequent real-world environmental monitoring tasks.

## 7. Conclusions

This study designed an MSA—UGAN model for the spatial and spectral fusion of S2 MSI and S3 OLCI images. Through the synergistic constraints of the network architecture and a hybrid loss function, the model effectively enhances the quality of the fusion results, along with the spatial and spectral fidelity required for water quality retrieval tasks. Comparative experiments demonstrate the HRMS images generated by MSA-UGAN provide superior texture and feature visual representation by restoring more high-frequency details. The model also outperforms traditional spatial–spectral fusion methods, SRCNN, and UCGAN across multiple quantitative evaluation metrics. To further verify the potential application of MSA-UGAN-generated fused images in lake water quality monitoring, this study used the best-performing machine learning model to generate spatial distribution maps of Chl-a concentration in Chen Lakes. Furthermore, the spectral advantages of the fused images were further validated through the synergistic inversion of spectral reflectance data from S2 MSI images.

The results of Chl-a concentration inversion based on the performance-optimized RF inversion model indicated that the overall water quality of Chen Lake was in a transitional stage between mesotrophic and mildly eutrophic conditions. Spatially, elevated Chl-a concentrations are primarily observed in the southwestern nearshore waters, which is likely attributable to local aquaculture and agricultural activities. Therefore, for future eutrophication control and wetland restoration, the southwestern nearshore zone should be regarded as a priority management area, where targeted and refined strategies are recommended.

## Figures and Tables

**Table 1:** Detailed information on the experimental dataset. Sa represents spatial resolution, and Se represents spectral resolution.

Data		Sa	Se	Training Patches: 2048	Testing Patches: 62
Input	S2_S3_SRF_	60 m	21_SRF_	320 × 320 × 21_SRF_	320 × 320 × 21_SRF_
	S3 OLCI	300 m	21	64 × 64 × 21	64 × 64 × 21
Output		60 m	21	320 × 320 × 21	

**Table 2:** Results of the ablation study on the newly added loss term conducted on the dataset created in this study. The last row represents the ideal value for each metric. ‘√’ represents that *L_DCSA_* or the combination of four losses has been added in the experiment.

Loss Term		Non-Reference Metrics			Reference Metrics		
*L_rec_* + *L_adv_* + *L_cyc_* + *L_nrf_*	*L_DCSA_*	D_λ_	D_S_	QNR	SAM	ERGAS	SSIM
√		0.0327	0.0391	0.9483	1.6975	2.2233	0.8756
√	√	0.0252	0.0389	0.9709	1.1331	2.2734	0.9087
Ideal Value		0	0	1	0	0	1

**Table 3:** Results of parameter sensitivity testing for the newly added loss term on the dataset created in this study. The last row indicates the ideal value for each metric. The optimal weight of the newly added loss term, namely **λ_4_**, is highlighted in bold.

Weight					Non-Reference Metrics			Reference Metrics		
λ_1_	λ_2_	λ_3_	λ_4_	λ_5_	D_λ_	D_S_	QNR	SAM	ERGAS	SSIM
0.0005	0.001	0.001	0.1	1	0.0289	0.0389	0.9675	1.1425	2.2762	0.9073
0.0005	0.001	0.001	**1**	1	0.0252	0.0389	0.9709	1.1331	2.2734	0.9087
0.0005	0.001	0.001	10	1	0.0255	0.0406	0.9688	1.1409	2.2741	0.9069
Ideal Value					0	0	1	0	0	1

**Table 4:** Quantitative results of the comparative experiments. Bold font signifies the optimal performance. The final score for each metric is the average of all test samples and will be reported.

Type	Model	Non-Reference Metrics			Reference Metrics		
		D_λ_	D_S_	QNR	SAM	ERGAS	SSIM
Tradition methods	GS	0.0429 ± 0.0559	0.2668 ± 0.0348	0.7037 ± 0.0504	1.8377 ± 0.3102	3.1661 ± 0.4718	0.8507 ± 0.0305
	SFIM	0.0740 ± 0.0507	0.1468 ± 0.0351	0.7921 ± 0.0580	2.0445 ± 0.4243	4.6229 ± 0.5588	0.7934 ± 0.0381
	MTF_GLP	0.0926 ± 0.0595	0.1697 ± 0.0355	0.7560 ± 0.0539	2.1143 ± 0.5257	3.6891 ± 0.5636	0.7825 ± 0.0291
Supervised	SRCNN	0.0453 ± 0.0288	0.0475 ± 0.0320	0.9227 ± 0.0425	1.6410 ± 0.2243	2.6026 ± 0.4436	0.8841 ± 0.0244
Unsupervised	UCGAN	0.0389 ± 0.0237	0.0452 ± 0.0213	0.9358 ± 0.0265	1.7577 ± 0.2562	**2.1749 ± 0.3641**	0.8964 ± 0.0249
	MSA-UGAN	**0.0252 ± 0.0129**	**0.0389 ± 0.0132**	**0.9709 ± 0.0241**	**1.1331 ± 0.2162**	2.2734 ± 0.3951	**0.9087 ± 0.0120**
Ideal Value		0	0	1	0	0	1

**Table 5:** Metrics are calculated on the fused and the S3 OLCI images. Metrics that need to be maximized are noted (↑), with the best value being 1. Metrics that need to be minimized are noted (↓), with the best value being 0.

Metrics	CC (↑)	RMSE (↓)
Mean	0.9998	0.0250
Maximum	0.9999	0.0429
Minimum	0.9991	0.0113
Median	0.9998	0.0225
Standard Deviation	0.0001	0.0100

**Table 6:** Pearson correlations between Chl-a concentration and spectral bands. The strength of the correlation is determined by the absolute value of the coefficient (|*r*|), where |*r*| ≥ 0.6 indicates a strong correlation. All strong correlations are highlighted in bold in the table.

Data	Variables	*r*	Variables	*r*	Variables	*r*
Fused Image	**Oa3**	**−0.63**	**Oa8**	**−0.68**	**Oa6/Oa8**	**0.78**
	Oa4	−0.59	Oa10	−0.53	**Oa11/Oa8**	**0.81**
	Oa5	0.31	**Oa11**	**0.71**	**(Oa11 − Oa8)/(Oa11 + Oa8)**	**0.85**
	Oa6	0.52	Oa12	0.45	**Oa11 − Oa8**	**0.62**
	Oa7	0.40	Oa13	0.21	Oa10 − Oa8	0.57
MSI Image	**B1**	**−0.61**	**B5**	**0.69**	B8A	−0.18
	B2	−0.54	B6	0.54	**B5/B4**	**0.73**
	**B3**	**0.68**	B7	0.29	**B5/B1**	**0.78**
	B4	−0.51	B8	0.11	**(B5 − B4)/(B5 + B4)**	**0.81**

**Table 7:** Results of hyper-parameter optimization for each machine learning algorithm. Metrics that need to be maximized are noted (↑), with the best value being 1. Metrics that need to be minimized are noted (↓), with smaller values denoting a better model fit.

Algorithm	Hyper-Parameter	Ranges	Fused Image			S2 MSI Image		
			Optimal Value	R^2^ ↑	RMSE ↓ (µg/L)	Optimal Value	R^2^ ↑	RMSE ↓ (µg/L)
RF	Max depth	[1, 10]	4	0.85	3.26	5	0.71	3.80
	Min samples leaf	[1, 10]	2			2		
	n_ estimators	[1, 500]	40			70		
XGBoost	Max depth	[1, 10]	3	0.81	3.39	3	0.70	3.81
	Learning rate	[0.01, 0.1]	0.05			0.02		
	n_ estimators	[1, 500]	80			80		
SVR	Kernel functions	poly, rbf, sigmoid	rbf	0.73	3.78	rbf	0.61	4.27
	C	[0.1, 10]	0.8			1		
	Gamma	[0.1, 1]	0.5			0.5		

**Table 8:** Performance comparison of Chl-a concentration inversion models. ↑ and ↓ both represent the optimal direction. The ideal optimal value of R^2^ is 1. Lower values of MAE and RMSE indicate higher inversion accuracy.

Water Quality Parameter	Data	Algorithms	R^2^ ↑	RMSE ↓ (µg/L)	MAE ↓ (µg/L)
Chl-a	Fused Image	RF	0.87	3.17	2.29
		XGBoost	0.85	3.24	2.35
		SVR	0.76	3.67	2.51
	MSI Image	RF	0.75	3.70	2.51
		XGBoost	0.72	3.76	2.54
		SVR	0.63	4.22	2.93

## Data Availability

The data presented in this study are available on request from the corresponding author. The data are not publicly available because they were provided by a government department, and the government department requires confidentiality.
